# IL-10 Reduces Levels of Apoptosis in *Toxoplasma gondii-*Infected Trophoblasts

**DOI:** 10.1371/journal.pone.0056455

**Published:** 2013-02-13

**Authors:** Mingdong Zhao, Ruijin Zhang, Xiaoyan Xu, Yang Liu, Haixia Zhang, Xiaoyu Zhai, Xuemei Hu

**Affiliations:** 1 Department of Immunology, Binzhou Medical University, Yantai, People's Republic of China; 2 Department of Radiology, Yantai Affiliated Hospital of Binzhou Medical University, Yantai, People's Republic of China; Charité, Campus Benjamin Franklin, Germany

## Abstract

**Background:**

To analyze the effects of IL-10 on the HLA-G expression and the apoptosis of trophoblasts infected with *Toxoplasma gondii*.

**Methods:**

*T. gondii*-infected or uninfected human trophoblasts and immortalized human placental BeWo cells were cultured with or without human IL-10. Uninfected and infected cells without IL-10 cells served as controls. HLA-G expression was measured by real-time PCR and flow cytometry, respectively. Cells apoptosis were analyzed by flow cytometry. Apoptosis associated moleculars were measured by real-time PCR and Western bolt.

**Results:**

HLA-G expression was increased in the infected trophoblasts and BeWo cells compared to uninfected cells. Treatment of infected cells with IL-10 decreased HLA-G expression compared to infected cells while no change in treatment of uninfected cells compared with uninfected cells. Levels of apoptosis and apoptosis associated caspase-3 and caspase-8 decreased and c-FLIP levels increased in treated infected cells with IL-10 compared to infected cells and no difference in IL-10 treated uninfected cells compared to uninfected cells.

**Conclusions:**

IL-10 regulates HLA-G expression in *T. gondii*-infected trophoblasts. IL-10 treatment of infected trophoblasts reduced levels of apoptosis. This may contribute to the improvement in pregnancy outcomes when women infected with *T. gondii* treated with IL-10.

## Introduction

*Toxoplasma gondii* (*T. gondii*) is an obligate intracellular protozoan. Infection with *T. gondii* is generally benign, but early maternal infection may result in severe congenital toxoplasmosis via vertical transmission and may result in miscarriage, stillbirth, or fetal abnormalities [1], [2]. At the maternal-fetal interface, multiple immune mechanisms, including the Th1/Th2 balance, participate in induction of immune tolerance that protects the embryo from maternal attack. IL-10, as a key regulator of Th2 immune responses, down-regulates Th1 response-driven inflammatory reactions, preventing inflammatory damage during parasite infection [3]. We recently demonstrated that when pregnant C_57_BL/6 mice infected with *T. gondii* were treated with recombinant mouse IL-10 fewer abnormal pregnancy outcomes were observed compared with untreated infected mice, possibly due to correction of the Th1/Th2 balance [4].

HLA-G, a non-classical HLA-I molecule expressed in human trophoblast cells, can prevent the maternal attack on embryonic antigens by abrogating the activity of maternal natural killer (NK) cells against fetal tissue [5]. HLA-G induces expression of Th2-type cytokines and inhibits expression of Th1-type cytokines such as TNF-α and IFN-γ [6], [7]. HLA-G expression in human primary trophoblasts is influenced by *T. gondii* infection, and HLA-G expression in trophoblast cells is up-regulated by treatment with IL-10 [8]. We demonstrated that expression of the mouse ortholog of human MHC I b, Qa-1, on the trophoblasts is up-regulated by treatment of cells with IL-10 [4]. However, the relationship between IL-10 and the HLA-G expression in *T. gondii*-infected trophoblast cells and the roles of these molecules in pregnancy during *T. gondii* infection are not clear.

The induction of apoptosis and necrosis of trophoblasts may be one of the direct reasons for abnormal pregnancy in women infected with *T. gondii*. As an important Th2-type cytokine, IL-10 reportedly decreases spontaneous monocyte apoptosis via up-regulation the FLICE inhibitory protein FLIP [9] and down-regulation of caspase-3 activity in human articular chondrocytes *in vitro* [10]. Here we tested our hypothesis that HLA-G expression in and apoptosis of trophoblast cells infected with *T. gondii* are influenced by IL-10, which may counteract the abnormal pregnancy outcomes elicited by *T. gondii* infection.

## Methods

### Isolation and purification of human trophoblast cells

Tissues were obtained following elective pregnancy termination performed at 6∼8 weeks of pregnancy in Yantai Chinese Medicine Hospital. The protocols used in this study were approved by University of Binzhou Medical College Ethics Committee, and informed consent was obtained from all patients. The villi were immediately washed with cold phosphate buffered saline (PBS) and cut into 1 to 3 mm^3^ fragments. These samples were digested with 0.25% trypsin (Sigma-Aldrich) and 0.02% deoxyribonuclease I (Sigma-Aldrich) three times for 30 min each at 37°C with constant shaking. The dispersed trophoblast cells were filtered through a 200-µm nylon gauze and were loaded onto a discontinuous Percoll gradient of 25–65% (GE Healthcare), followed by centrifugation at 2000 rpm for 20 min to separate different cell types. Cells between the density markers of 1.048 and 1.062 g/ml were collected, washed twice with Hank's solution, equilibrated at 37°C, and cultured for 1 hr in high-glucose, phenol red free DMEM (Hyclone) containing 20% fetal bovine serum (FBS, Gibco Co.), 2.5 mM L-glutamine, 15 mM HEPES, 100 units/ml penicillin, and 100 mg/ml streptomycin. The the culture suspension was transferred to culture flasks coated with matrigel (BD Biosciences; matrigel/DMEM, 1∶2). The cultures was incubated at 37°C in 5% CO_2_ and saturated humidity.

### BeWo cell culture

BeWo cells, used as experimental model of trophoblast cells (B. F. Barbosa, 2008) [11] in this study, were kindly provided by Institute of Gynecology and Obstetrics of Fudan University. The cells were maintained with DMEM/F12 (Hyclone) medium containing 10% FBS (Gibco Co.) in a flask (approximate 4×10^5^ cells). The medium was changed every other day, and cells were incubated at 37°C in 5% CO_2_ and saturated humidity.

### Infection and co-culture with IL-10

*T. gondii* expressing Yellow Fluorescent Protein (YFP-*T. gondii*, which was RH strain, belongs to type I strain)), a gift from Dr. Boris Striepen of the Tropical and Emerging Global Diseases Center, Georgia University , USA, were maintained by passage once every 54 hr in the peritoneal fluid from Kunming mice executed by cervical dislocation following anesthesia. BeWo cells were cultured on 12.5-mm flask (4×10^5^ cells/flask/2 ml) for 24 hr at 37°C and 5% CO_2._ Cells were washed with PBS and infected with *T. gondii* RH strain at the ratio of 3∶1 (parasite∶cell). Recombinant human IL-10 (purchased from Peprotech) was added to non-infected cells after 1 hr infected with *T. gondii* and at the same time, IL-10 was added to uninfected cells for 16 hr, 24 hr, 36 hr, 48 hr and 60 hr, respectively at a concentration of 50 ng/ml. Cultures was maintained as described above. This study was carried out in strict accordance with the recommendations in the Guide for the Care and Use of Laboratory Animals of Binzhou Medical University. The protocol was approved by the Committee on the Ethics of Animal Experiments of Binzhou Medical University.

### HLA-G expression analysis

Single-cell suspensions of trophoblasts or BeWo cells were prepared by digestion with 0.25% trypsin containing 0.04% EDTA. Cells were washed with PBS and then incubated with 20 µl anti-HLA-G-PE monoclonal antibody (eBioscience) in the dark for 30 min at 4°C. After washing twice with PBS, the cells were resuspended and subjected to four-color FACS on a BD flow cytometer. Data were analyzed using Cell Quest software (BD Biosciences). HLA-G mRNA expression was analyzed with real-time RT-PCR after total RNA extraction and reverse transcription.

### Apoptosis analysis

Cells (2×10^5^cells in 100 µl) were washed with annexin-binding buffer followed by incubation with 1 µl annexin V-PE and 5 µl propidium iodide (KeyGEN Biotech) for 15 min at room temperature in the dark. Cells were then washed with binding buffer and subjected to four-color FACS.

### Caspase-3, caspase-8, and c-FLIP analyses

Total RNA was extracted from BeWo cells and reverse transcribed into cDNA using oligo (dT) primers and RNase H-minus reverse transcriptase (Toyobo) according to the manufacturer's instructions. Primers were designed using Primer Premier 5.0 (Premier Biosoft Inc.). Primers sets for c-FLIP_L_, c-FLIP_S_, caspase-8, caspase-3, and β-actin are listed in Table 1. Amplification was carried out in a PCR mix containing 2 µl first strand cDNA (diluted 10 fold) as a template, 9.5 µl ddH_2_O, 12.5 µl SYBR Green mix (Fermentas), 0.5 µl 20 pmol/µl each primer (Shanghai Sangon Biotech Co.). Thermocycling was performed at 95°C for 5 min, 95°C for 15 s, annealing at 58°C or 63°C for 15 s, and extension at 72°C for 45 s for 40 cycles. Specific PCR products were quantified by melting curve analysis and visualized by agarose electrophoresis. Values of all genes were normalized to the levels of the housekeeping gene β-actin. Gene expression levels were expressed as fold increases relative to control by the 2-(Ct method.

**Table 1:** Primer sequences and product lengths.

Name	Sequences (5′-3′)	
HLA-G (human)	Forward primer	CTGACCCTGACCGAGACCTGG
	Reverse primer	GTCGCAGCCAATCATCCACTGGAG
c-FLIPs (human)	Forward primer	GGACCTTGTGGTTGAGTTGG
	Reverse primer	ATCAGGACAATGGGCATAGG
c-FLIPL (human)	Forward primer	GGCTCCCAGAGTGTGTATGG
	Reverse primer	AGCTTCTCGGTGAACTGTGC
caspase-8 (human)	Forward primer	TCCTGCCTGCCTGTACCCCG
	Reverse primer	GCCCAACCTCACGTGCCCAG
caspase-3 (human)	Forward primer	GACTGTGGCATTGAGACAGAC
	Reverse primer	CTTTCGGTTAACCCGGGTAAG
β-actin (human)	Forward primer	TTGTTACAGGAAGTCCCTTGCC
	Reverse primer	ATGCTATCACCTCCCCTGTGTG

### Western blot analysis

Cells were harvested, washed twice with ice-cold PBS, suspended in lysis buffer (Biyuntian, China), and incubated on ice for 30(min. The cell lysates were then centrifuged at 4(C at 12000(rpm for 10(min, and an appropriate amount of supernatant (determined by protein assay) was mixed with 5× SDS-PAGE loading buffer, and was boiled to denature at 95(C for 10 min. The denatured protein samples were fractionated on 10% SDS-PAGE (polyacrylamide gel electrophoresis gels) (Biyun tian, China), transferred onto a PVDF membranes (BD, pharMingen, San Jose, CA) and incubated with 5% nonfat dry milk in Tris-buffered saline/0.2% Tween-20 for blocking 2 hr at room temperature. The membranes were incubated with mouse anti-human caspase3 (BD Manufacturer, USA), caspase8, active-caspase8 (both from eBioscience, USA), c-FLIPs, c-FLIPL antibodies (both from Millipore, USA); rabbit anti-active3 antibody (Boshide, China) at 4(C over night. Membrans were then washed (3×15 min) with 1× TBS-T followed by incubation with horseradish peroxi-dase–conjugated goat anti-mouse and goat anti-rabbit IgG secondary antibodies (1∶5,000 dilution; Amersham Biosciences) for 1 hr at room tempreture. The membranes were again washed five times in 1× TBS-T and proteins were visualized using an enhanced chemiluminescence kit (ECL; Roche Diagnostics). The bolt was then exposed to film for various lengths of time. The GAPDH immunoblot using rabbit anti-GAPDH polyclonal antibody (Santa Cruz Biotechnology) was incubated as control to demonstrate equal loading.

### Statistical analysis

Data are presented as the mean ± S.E.M. Statistical analyses were performed using SPSS 13.0 statistical software version. One-way ANOVA was used for comparing the three independent groups at each time point. A *p* value less than 0.05 was considered significant, and a *p* value less than 0.01 was considered very significant.

## Results

### *T. gondii* infection of trophoblast and BeWo cells

**Figure 1:**
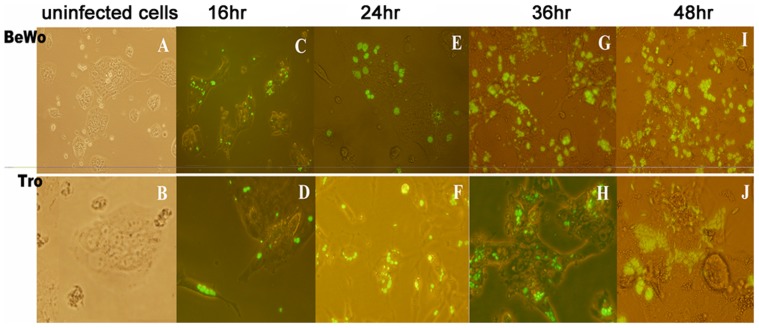
Fluorescence analysis of infection of BeWo cells and trophoblasts with YFP-*T. gondii*. Infected and uninfected (A) BeWo cells and (B) trophoblasts were observed under fluorescence microscope. The infection with *T. gondii* expressing yellow fluorescent protein in BeWo and trophoblasts were observed by fluorescence microscopy at (C, D) 16 hr, (E, F) 24 hr, (G, H) 36 hr, and (I, J) 48 hr. At 16 hr, coupled or ternate tachyzoites were observed inside the cells. Tachyzoites arranged in a chrysanthemum shape were observed at 24 hr. Broken cells and scattered tachyzoites were observed at 48 hr.

Infection of trophoblasts and BeWo with *T. gondii* tachyzoites was detected due to the presence of yellow fluorescence spots inside cells by fluorescence microscopy (Figure 1). At 16 hr post infection, coupled or ternate tachyzoites were observed inside the cells. Tachyzoites arranged in a chrysanthemum shape in parasitophorous vacuoles were observed at 24 hr in both cell types and increased with time. Lysed cells and scattered tachyzoites were observed in the culture at 48 hr.

### HLA-G expression increases upon infection and decreases with IL-10 treatment

**Figure 2:**
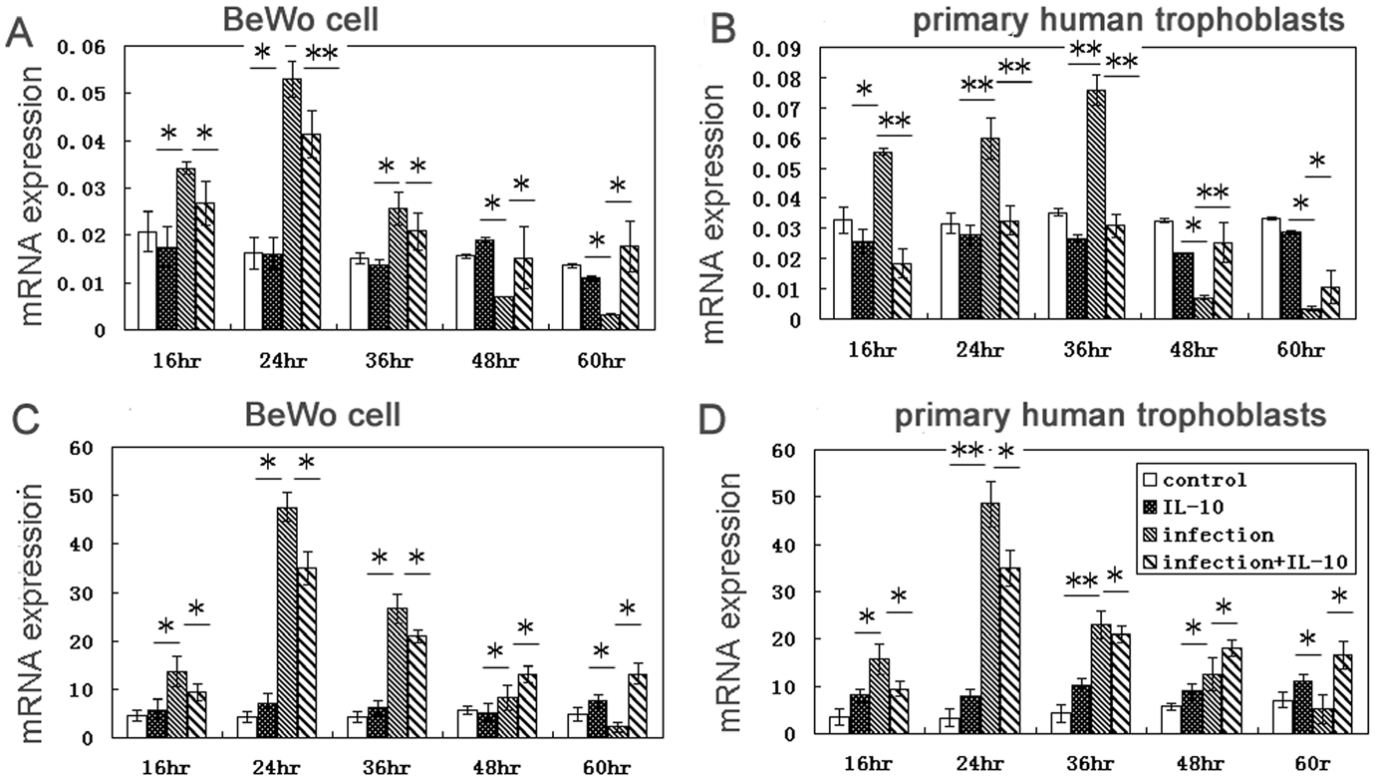
HLA-G expression in infected cells decreased by IL-10 treatment. HLA-G mRNA expression was analyzed in infected and uninfected BeWo cells (A) and primary human trophoblast cells (B) with and without IL-10 treatment. (C) HLA-G protein expression was analyzed in infected and uninfected BeWo cells with and without IL-10 treatment. (D) HLA-G protein expression was analyzed in infected and uninfected trophoblasts with and without IL-10 treatment. * *p*<0.05; ** *p*<0.01.

Real-time PCR analysis showed that HLA-G mRNA levels were higher in infected cells than uninfected BeWo cells (Figure 2A) and human primary trophoblast cells (Figure 2B). Levels of HLA-G protein were higher in infected BeWo cells (Figure 2C) and trophoblast cells (Figure 2D) than in uninfected cells. But their was no difference in the levels of HLA-G with IL-10 treated uninfected cells compared to uninfected cells. When infected cells were treated with IL-10, a decreased in levels of HLA-G protein compared to infected, untreated cells was observed at 16, 24, and 36 hr. We observed higher levels of HLA-G in treated, infected cells than in untreated, infected cells at 48 hr and 60 hr.

### *T. gondii* –induced apoptosis inhibited by IL-10 treatment

**Figure 3:**
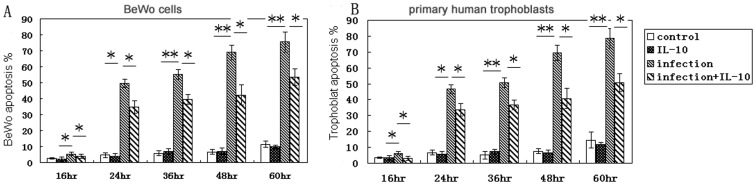
*T. gondii* infection induces apoptosis. Apoptotic cells were detected by flow cytometry after annexin V-PE/PI staining. PE^+^PI^−^ and PE^+^PI^+^ cells were defined as apoptotic. The percentage of apoptotic cells was lower in infected cells treated with IL-10 than in infected cells without IL-10 treated while there was no difference between uninfected cells and IL-10 treated uninfected cells. * *p*<0.05, ** *p*<0.01.

**Figure 4:**
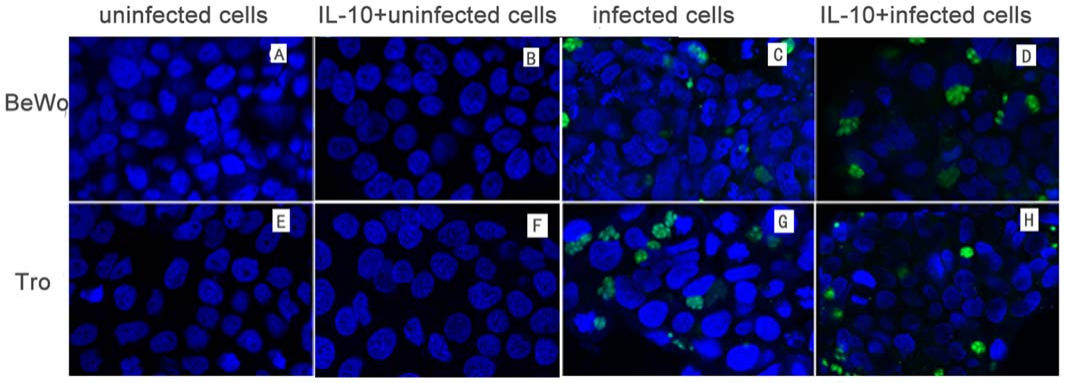
Hoechst 33258 staining of BeWo cells and trophoblasts at 24 hr after challenge with *T. gondii*. (A) Uninfected BeWo cells, (E) uninfected trophoblasts, (B) uninfected BeWo cells treated with IL-10, (F) uninfected trophoblasts treated with IL-10, (C) infected BeWo cells, (G) infected trophoblasts, (D) infected BeWo cells treated with IL-10, and (H) infected trophoblasts treated with IL-10 were fixed, stained with Hoechst 33258, and observed by microscopy. Apoptosis was indicated by nuclei fragmentation or crescent shaped nuclei. Most of apoptotic nuclei were at a distance from the parasitophorous vacuoles (PV) and nuclei close to parasitophorous vacuoles usually showed normal appearances. The results also showed that the apoptotic cells increased in infected cells compared to uninfected cells and decreased with IL-10 treatment while there was no significantly difference between IL-10 treated uninfected cell and uninfected cells.

*T. gondii* induced apoptosis in both trophoblast and BeWo cells as shown by annexin-V-PE/PI staining. Apoptosis of infected cell was obvious at 16 hr after infection and increased with the infection time. The number of apoptotic infected cells decreased following IL-10 treatment compared to infected cells while their was no difference between uninfected cells and IL-10 treated uninfected cells (Figure 3). We examined BeWo cells and trophoblast cells cultures at 24 hr after challenge for proximity of obvious vacuoles to distorted (indented) nuclei stained with Hoechst 33258. At 24 hr post infection, most nuclei directly adjacent to parasitophorous vacuoles were not distorted while far away from the infected cells were distorted badly. In addition, the abnormal nucleus in IL-10 treated infected cells decreased compared to infected cells, while IL-10 had no role in decreasing the uninfected cells without *T. gondii* infection (Figure 4), suggesting that IL-10 treatment of cells in infected groups is associated, at least initially, with resistance to *T. gondii*–stimulated apoptosis in the cultures.

**Figure 5:**
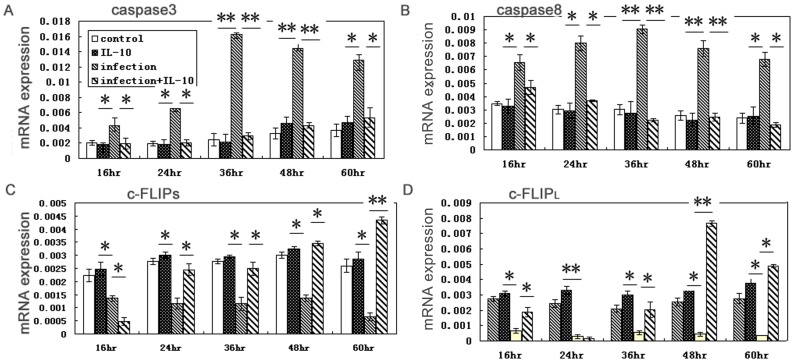
Changes in levels of mRNAs encoding caspase-3, caspase-8, c-FLIP_L_ and c-FLIP_S_ in BeWo cells due to infection are reverted upon IL-10 treatment. Caspase-3 (A) and caspase-8 (B) were both up-regulated while c-FLIP_S_ (C) and c-FLIP_L_ (D) were both down-regulated in infected cells compared to uninfected cells. Levels were reduced in infected cells treated with IL-10 compared to infected cells while no changes in treatment IL-10 in untreated cells relative to uninfected cells..IL-10 treatment solely modulates excessive-expression of apoptosis associated cells and no role in normal expression. * *p*<0.05; ** *p*<0.01.

**Figure 6:**
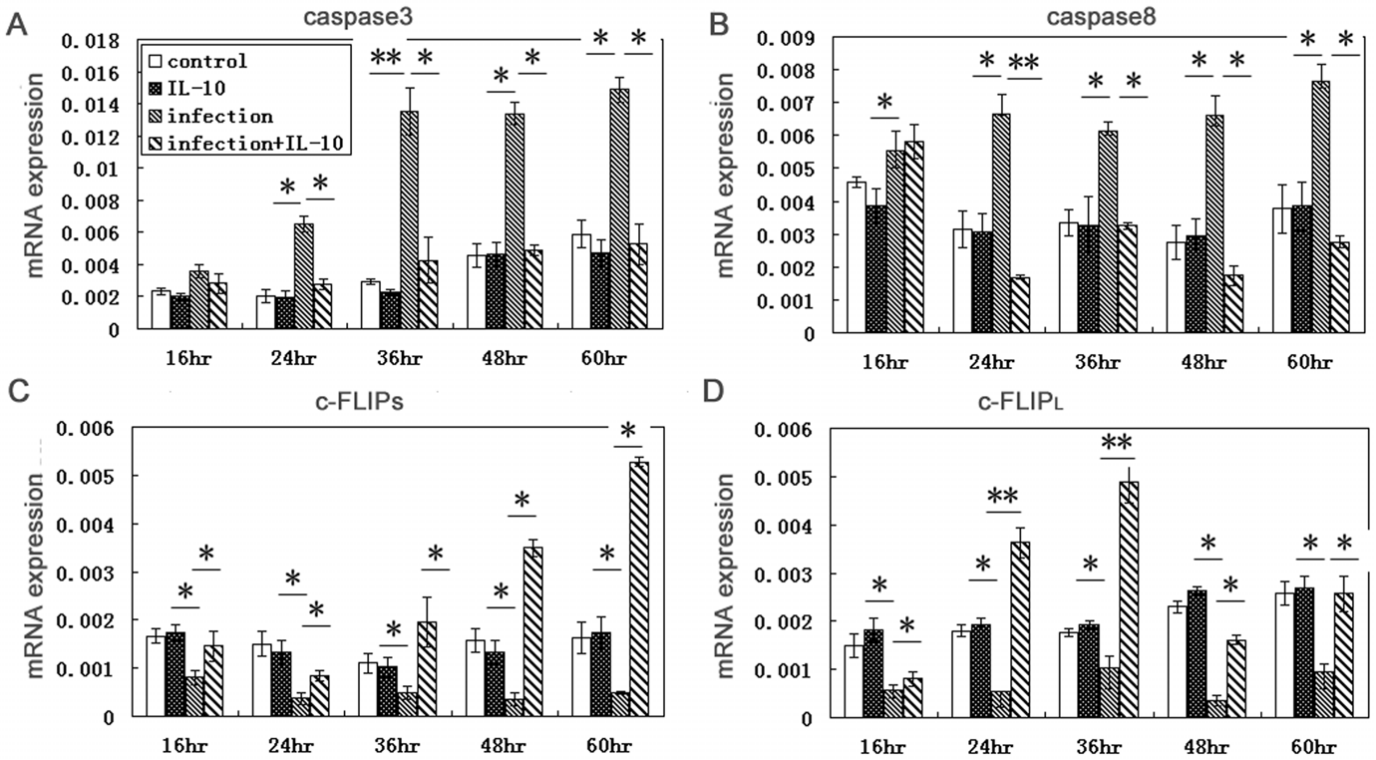
Changes in expressions of mRNAs encoding caspase-3, caspase-8, c-FLIP_L_ and c-FLIP_S_ in primary human trophoblast cells result from infection are reverted upon IL-10 treatment. Caspase-3 (A) and caspase-8 (B) were both increased while c-FLIP_S_ (C) and c-FLIP_L_ (D) were decreased in infected cells relative to uninfected cells. Levels were reduced in infected cells treated with IL-10 compared to infected cells while no changes in treatment IL-10 in untreated cells relative to uninfected cells. * *p*<0.05; ** *p*<0.01.

To analyze the key molecules involved in apoptosis, we evaluated levels of caspase-3, caspase-8, and c-FLIP mRNAs expression in BeWo cells (Figure 5) and primary human trophoblast cells (Figure 6). In infected cells, c-FLIP_L_ and c-FLIP_S_ mRNA levels were decreased and caspase-3 and caspase-*8* levels were increased relative to uninfected cells. In infected cells treated with IL-10, c-FLIP_L_ and c-FLIP_S_ expression was higher and caspase-8 and caspase-3 levels were lower than in infected cells, while the three molecules above have no significantly difference in IL-10 treated uninfected cells compared with uninfected cells.

**Figure 7:**
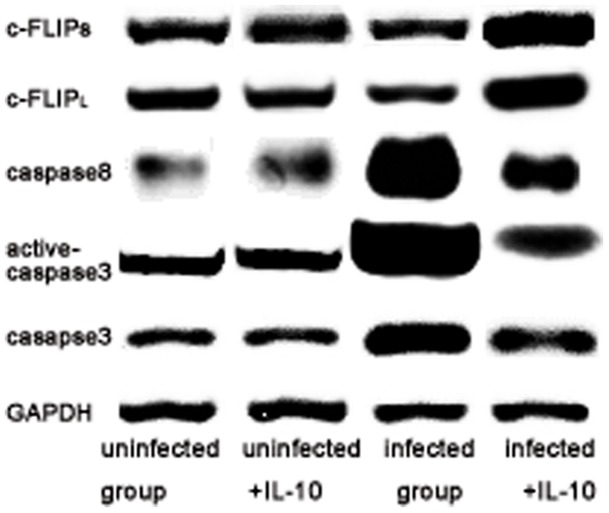
Caspase-3, active caspase-3, caspase-8, active-8, c-FLIP_S_ and c-FLIP_L_ in human trophoblast cells were also analyzed by Western blot. The results showed that caspase-3, active caspase-3 and caspase-8 were up-regulated in infected cells compared to uninfected cells while down-regulated in IL-10 treated cells compared with infected cells (P<0.05). In this study, levels of active caspase-8 could not be quantified in both infected cells and uninfected cells. In addition, down-regulation of c-FLIP_S_ and c-FLIP_L_ expression occurred in infected cells relative to uninfected cells and up-regulation in IL-10 treated infected cells (P<0.05). Interestingly, it was also no difference between uninfected cells and IL-10 treated uninfected cells (P>0.05).

Changes of caspase-3, caspase-8, and c-FLIP were detected using Western blot to deduce if apoptosis associated molecules involved in trophoblast cells apoptosis with *T. gondii* infection. As shown in Figure 7, the results in infected cells showed that c-FLIP_L_ and c-FLIP_S_ were down-regulated while caspase-3, active-caspase-3 and caspase-8 levels were up-regulated compared with uninfected cells. Compared with infected cells, c-FLIP_L_ and c-FLIP_S_ were higher, but caspase-8, caspase-3 and active-caspase-3 were lower than IL-10 treated infected cells. There were no significantly difference between uninfected cells and IL-10 treated uninfected cells.

### Correlation Analysis

The spearman's correlation analysis was used to quantitate the correlation between the HLA-G expression and apoptosis of human primary trophoblast cells and BeWo cells at 24 hr infection. The apoptosis of trophoblast cells and BeWo cells correlated positively with HLA-G at 24 hr infection of *T. gondii* (r = 0.733, P = 0.0142; r = 0.814, P = 0.0084).

## Discussion

Expression of HLA-G is up-regulated in cells infected with HIV and neurotropic viruses and in damaged cells and tissues by microenvironmental factors including cytokines such as IL-10 and TNF-α [12], [13]. Trophoblasts produce TNF-α [14], [15], and HIV-infected trophoblast cells secrete high levels of IFNs [16]. These pro-inflammatory cytokines may be involved in HLA-G up-regulation, and HLA-G may allow infected cells or tumor cells to escape immune responses [16]. *T. gondii* tachyzoites enter almost all nucleated mammalian cells [17] and cause adverse outcomes in pregnancies of both humans and mice. Trophoblasts, an important source of *T. gondii* vertical transmission, undergo apoptosis or necrosis when the parasitophorous vacuole ruptures. In this study, we found that both human primary trophoblast cells and BeWo cells could be productively infected by *T. gondii* RH strain easily and obvious apoptosis resulted. This result was different from what M. B. Angeloni et al showed that RH strain infected BeWo cells occurred a lower apoptosis index than non-infected controls at 2 hr, 6 hr and 12 hr infection [11]. Another study showed that in the early stage of infection, *T. gondii* resisted infected cells apoptosis in order to proliferate itselves while in the later stage of infection. *T. gondii* may promote cells apoptosis which were distance from the parasitophorous vacuoles to prevent *T. gondii* from spreading around [18]. In our study, we observed cells of apoptosis increased in the later stage of infection at 16 hr, 24 hr, 36 hr, 48 hr, and 60 hr, actually this result was paralleled with what previous studies showed.

In our study, HLA-G was strongly up-regulated at 24 hr following *T. gondii* infection of primary human trophoblasts and of immortalized BeWo cells, likely a consequence of pro-inflammatory cytokines secretion in response to the parasite that allows *T. gondii* to subvert host defenses during the tachyzoite multiplication period. When infected cells were treated with IL-10, HLA-G expression in human trophoblast cells was significantly down-regulated compared with levels in the infected cells. The down-regulation of HLA-G by IL-10, an anti-inflammatory cytokine, may be due to the suppression of pro-inflammatory cytokine secretion by trophoblasts. While HLA-G expression in IL-10 treated uninfected cells has no difference from uninfected cells without IL-10 treatment. This result showed that IL-10, as a negative regulated factor, may mainly modulate the expression of HLA-G. Interestingly, this down-regulation of HLA-G in the treated infected cells was observed at 36 and 60 hr post infection and then HLA-G expression was up-regulated compared with the untreated infected group; this is consistent with the data that indicates that IL-10 induces HLA-G expression [19] and with results of our study *in vivo* [4]. The up-regulation of HLA-G in trophoblast cells by IL-10 at later stages of infection may enhance effects of inhibitory receptors expressed on decidual NK cells. It is likely that IL-10 plays roles both as an anti-inflammatory agent and in inducing HLA-G expression, which may be associated with the improvement of pregnancy outcomes in *T. gondii*-infected mice [4]. In addition, previous study showed that up-regulation of HLA-G could increase apoptosis of HTR8/svneo trophoblast cells [20]. In our study, similar result was showed that the apoptosis of BeWo cells and primary trophoblast cells were positively with the up-regulation of HLA-G expression; meanwhile, IL-10 treatment could cause a decrease of HLA-G in early stage of infection with *T. gondii*. These results demonstrated that the apoptosis decreased with IL-10 adding may be partly due to IL-10 induced up-regulation of HLA-G.

The cellular FLICE inhibitory protein, c-FLIP, an inhibitor of apoptosis, prevents the proteolytic activation of pro-caspase-8 by blocking caspase-8 recruitment by Fas associated death domain (FADD) [21], [22]. Therefore, in a pro-apoptotic state, one would expect to see a decrease in c-FLIP and, subsequently, an increase in caspase-8 and caspase-3 activities [23], [24]. We detected the long isoform c-FLIP_L_ and the short isoform c-FLIP_s_ in this study, and we found that *T. gondii* infection down-regulated levels of both c-FLIP isomers, accompanied by an up-regulation of caspase-8, caspase-3. It has been reported that high local concentrations of the pro-caspase zymogen within the DISC death-inducing signaling complex (DISC) leads to auto-processing and activation of caspase-8 [25]. In this study, result showed that active caspase-3 was increased in infected cells compared to uninfected cells. This result suggested *T. gondii* infection might also directly facilitate the caspase-3 recruitment and activation. However, active caspase-8 was not detected in every group. This result indicated that there was no obvious change of active-caspase-8 among four groups. Therefore, these results indicated that *T. gondii* induced BeWo and trophoblast cells apoptosis mainly via active caspase-3 pathway induced apoptosis.

We also have examined the effects of the recombinant human IL-10 on apoptosis of *T. gondii-*infected trophoblasts. Treatment with IL-10 significantly decreased *T. gondii-*induced apoptosis, up-regulated c-FLIP expression, and down-regulated caspase-8, caspase-3 and active caspase-3 expression. IL-10 may reduce apoptosis through an up-regulation of c-FLIP, which in turn prevents the proteolytic activation of pro-caspase-8 by blocking caspase-8 recruitment by FADD, leading to a down-regulation of caspase-3 activity. These results are in accordance with previous analyses of IL-10 effects on c-FLIP and caspase-8 in monocytes and mouse intestinal epithelial cells [25], [26], and results of a study indicating that IL-10 alters the c-FLIP/caspase balance in human dermal fibroblasts [27]. In addition, in our study results showed that IL-10 treatment mainly significantly down-regulated active-caspase-3, it may demonstrated that IL-10 may mainly regulate apoptosis of trophoblast cells induced by *T. gondii* infection via affecting the caspase3-active-caspase3 pathway.

Most of the apoptosis observed at 24 hr post-infection occurred in uninfected cells in the vicinity of *T. gondii*–infected cells as evidenced by apoptosis in nuclei at a distance from parasitophorous vacuoles. This observation is consistent with other reports [18], [28], [29]. This “bystander killing” occurs in trophoblast cultures infected with cytomegalovirus [30] in which infected cells release or express agents cytotoxic to neighboring cells. In this study, we found that IL-10 reduced the extent of apoptosis of uninfected cells in the vicinity of *T. gondii* infected cells at early infection stage (24 hr), but the detailed mechanisms involved as well as the effects on pregnancy of infected mice deserve further investigation.
